# Pseudopapillary Tumor of the Pancreas: A Rare Cause of Extrahepatic Portal Hypertension

**DOI:** 10.7759/cureus.15707

**Published:** 2021-06-17

**Authors:** Arkadeep Dhali, Elaina Pasangha, Christopher D'Souza, Anirban Hazra, Gopal Krishna Dhali

**Affiliations:** 1 Department of Gastrointestinal Surgery, School of Digestive and Liver Diseases, Institute of Post Graduate Medical Education and Research, Kolkata, IND; 2 Department of Critical Care Medicine, Narayana Hrudayalaya, Bangalore, IND; 3 Department of Critical Care Medicine, St. John's Medical College, Bangalore, IND; 4 Department of Radiology, St. John's Medical College, Bangalore, IND; 5 Department of Gastroenterology, School of Digestive and Liver Diseases, Institute of Post Graduate Medical Education and Research, Kolkata, IND

**Keywords:** solid pseudo-papillary tumor of the pancreas, extrahepatic portal hypertension, splenic vein thrombosis, distal pancreatectomy, splenectomy

## Abstract

A solid pseudopapillary tumor (SPT) of the pancreas is an uncommon neoplasm, characterized by a well-encapsulated mass, with low malignant potential. It occurs predominantly in young females. We present a case of SPT of the pancreas which presented with sinistral portal hypertension. Despite characteristic radiological findings due to its rarity, it may be missed to more common conditions like peptic ulcer disease. Delayed diagnosis can lead to complications like portal hypertension. To the best of our knowledge, in existing medical literature, SPT of the pancreas in males has rarely been described. In our case, we found that the tumor was causing extrahepatic portal hypertension which is also a very unique presentation of this tumor. Due to its vague clinical manifestations, definitive diagnosis is often a challenge hence requiring prompt investigations.

## Introduction

A solid pseudopapillary tumor (SPT) of the pancreas is an uncommon neoplasm of the pancreas. These patients are mostly clinically asymptomatic but may present with a gradually enlarging mass per abdomen or vague abdominal discomfort [1,2]. Features of obstruction may occur if adjacent viscera gets compressed. SPT of the pancreas can be detected in many imaging modalities, like USG, CT, and MRI, which help to differentiate it from other pancreatic lesions [3].

## Case presentation

A 17-year-old male presented with complaints of pain in the epigastrium for the last six months. It was insidious in onset, intermittent, dull aching type of pain, reached a peak within 30 minutes, persisted for three to four hours, non-radiating, not related to food intake or bowel habits, not associated with nausea, vomiting, sweating, and relieved with oral non-steroidal anti-inflammatory drugs (NSAIDs). Pain recurred every 10 to 15 days and it was associated with a history of jaundice for 30 days. There was no history of abdominal distension, fever, loss of weight and appetite, vomiting after food intake, trauma to the abdomen, blood in stool, altered bowel and bladder habits, or mass in the abdomen. There was no history of abdominal surgery or biliary instrumentation. No other relevant past medical or surgical history was elicited.

Physical examination revealed icterus. The abdomen was soft, non-tender, with no free fluid. A large non-tender, non-pulsatile, retroperitoneal mass of size approximately 20 cm x 15 cm, extending across epigastric, umbilical, and left hypochondrium was found. It had a smooth surface, ill-defined margins, firm consistency, and restricted mobility. All other systemic examinations were within normal limits.

**Figure 1:**
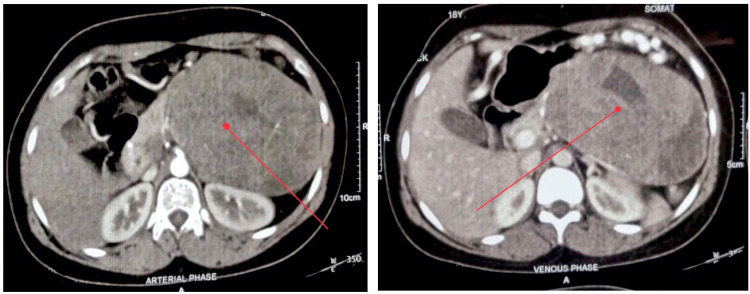
Contrast-enhanced CT abdomen showed well-defined, heterogeneously enhancing, soft tissue density lesion with areas of necrosis arising from the body and tail of the pancreas. The splenic vein was thrombosed with multiple perigastric collaterals.

**Figure 2:**
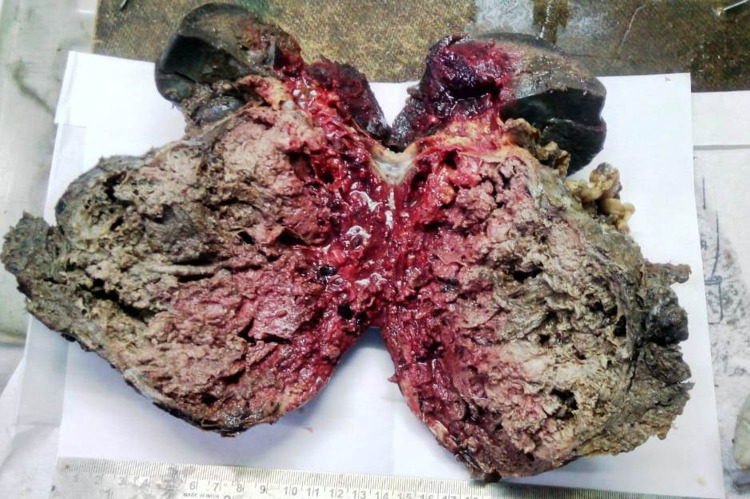
Gross specimen showing 25 cm x 20 cm x 10 cm cystic lesion related to the anteroinferior aspect of tail and body of pancreas along with the spleen.

**Figure 3:**
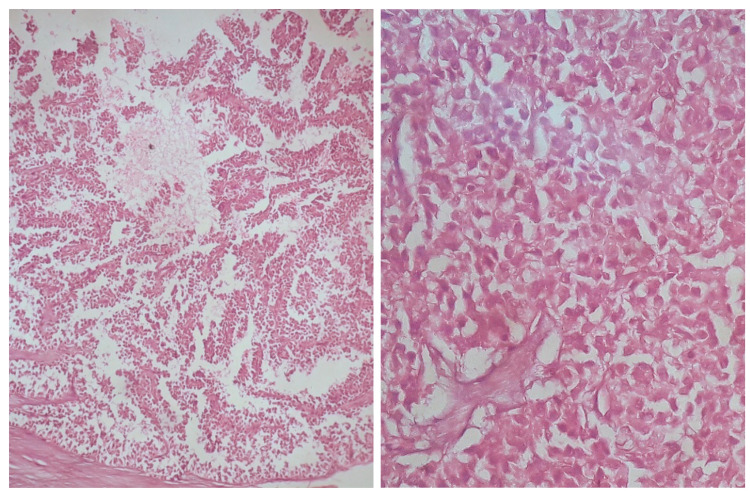
H&E image showing dyscohesive cells forming pseudo papillae around hyalinized stroma. The cells are round monomorphic and have eosinophilic to clear cytoplasm. Overall histological features are of a solid pseudopapillary tumor.

Laboratory investigations showed elevated serum bilirubin (3.2 g/dL), elevated serum transaminases (aspartate aminotransferase [AST]: 80IU/L, alanine aminotransferase [ALT]: 76IU/L, alkaline phosphatase [ALP]: 240IU/L), and reduced albumin (2g/dL). Blood counts and kidney function tests were normal. Serological markers for hepatitis B surface antigen (HBsAg)/anti-hepatitis C virus (Anti-HCV)/HIV were non-reactive. Autoimmune hepatitis markers and inflammatory markers like C-reactive protein (CRP) and procalcitonin were within the normal range. USG abdomen showed a large solid hypoechoic mass located at the tail of the pancreas. The splenic artery appeared to be engulfed by the mass. The splenic vein was thrombosed with large perigastric collaterals draining into the superior mesenteric vein. The celiac trunk and superior mesenteric artery appeared uninvolved. Contrast-enhanced CT abdomen (Figure 1) showed well-defined, heterogeneously enhancing, soft-tissue density lesion with areas of necrosis arising from the body and tail of the pancreas. The splenic vein was thrombosed with multiple perigastric collaterals. The rest of the study was normal. Upper GI endoscopy was normal. Cancer antigen 19-9 (CA 19-9) was 13 U/ml. Differentials considered at this point were chronic pancreatitis with a pseudocyst, acute pancreatitis sequelae with a pseudocyst, pancreatic tumor, and lipoma. In view of the above findings, the patient was taken up for distal pancreatectomy and splenectomy under general anaesthesia following informed consent. Operative findings showed a 25 cm x 20 cm x 10 cm cystic lesion related to the anteroinferior aspect of the tail and body of the pancreas, separated from the spleen, omentum, and gastrosplenic ligament (Figure 2). The pancreatic texture was soft. The tail of the pancreas was effaced. Left-sided portal hypertension present as evidenced by the dilated and tortuous gastroepiploic arcade. Abdominal drainage kit (ADK) drain fluid amylase level was 341 IU/l and Jackson-Pratt drain fluid amylase was 1809 IU/l on post-op day 3. On histopathological examination, a tumor was identified with solid and cystic areas with dyscohesive cells forming pseudo papillae around hyalinized stroma. The cells were round monomorphic and have eosinophilic to clear cytoplasm. Overall histological features were suggestive of an SPT (Figure 3). The resection margin showed no evidence of tumor involvement, and low-grade pancreatic intraepithelial neoplasia was observed in the peripancreatic parenchyma. No malignancy was found in the lymph nodes or the spleen. The post-operative period was uneventful and the patient was discharged on post-op day 10. Follow-up showed a decreasing trend of liver enzymes suggesting complete remission. Life-long needs for insulin and pancreatic enzyme supplementation if required were explained.

## Discussion

SPT was first reported in 1959 by Frantz [4]. It is described as an uncommon epithelial tumor of the pancreas which is 1-2% of non-endocrine tumors. Almost all SPTs harbor somatic point mutations in exon 3 of the β-catenin gene. Cyclin D1 is one of the targets of β-catenin which has a significant role in Wnt signaling in the tumorigenesis of solid-pseudopapillary neoplasm (SPN) [5].

Macroscopically, it is a round tumor with a thick fibrous capsule that grows outside the pancreas, and the cut surface shows a mixture of solid parts and necrotic or hemorrhagic cysts. Histologically, the tumor displays solid growth with hyaline stroma of acidophilic cells containing small round nuclei, while pseudopapillary or pseudorosette patterns are formed around vessels. It is frequently observed in young people and women with a male to female ratio of 1:10. This tumor has a predilection for young Asian and African-American women [2]. It is usually asymptomatic and only detected after growing as large as 2.5 to 10 cm in diameter. It affects the head, body, and tail of the pancreas with the same frequency. These patients are mostly clinically asymptomatic but may present with gradually enlarging mass or vague abdominal discomfort [1,2]. However, there have been no previous reports of SPN in patients presenting with jaundice and sinistral portal hypertension to the best of our knowledge although a few cases of serous cystadenoma of the pancreas associated with left-sided extrahepatic portal hypertension were previously reported [4,6,7]. There has been a case of SPT presenting with obstructive jaundice as well [2]. Most of the tumors are diagnosed with the help of ultrasound or CT scans, but MRI helps to delineate the hypervascular, round, encapsulated tumors with solid and cystic components. Endosonography provides fine needle aspiration (FNA) biopsy with the possibility of pre-operative pathologic diagnosis [8,9]. Due to its slow progression and asymptomatic beginnings, it is often diagnosed very late. It is often misdiagnosed to a more common diagnosis like pseudocyst of the pancreas. Although most tumors have low malignant potential, there is a propensity of this tumor to be locally aggressive invading the capsule and surrounding structures, mainly the spleen, portal vein, and duodenum. This will lead to splenic vein occlusion or left-sided extrahepatic portal hypertension. Despite the locally aggressive clinical features, the tumor has a low malignant potential and has a favorable prognosis, even in the presence of metastatic disease. Overall post-surgical treatment, five-year survival is as high as 97% [10].

## Conclusions

This is a unique case of SPT of the pancreas affecting a male patient. Here we report that SPT of the pancreas can cause complications like extrahepatic portal hypertension. One should suspect pancreatic diseases when coming across features of left-sided portal hypertension. Timely resection on diagnosis provides long-term survival. Histopathological examination of the sample stays as the investigation of choice as every pancreatic neoplasm behaves uniquely.
